# Expression of genes involved in progesterone receptor paracrine signaling and their effect on litter size in pigs

**DOI:** 10.1186/s40104-016-0090-z

**Published:** 2016-05-25

**Authors:** Xiao Chen, Jinluan Fu, Aiguo Wang

**Affiliations:** College of Animal Sciences and Technology, National Engineering Laboratory for Animal Breeding & Key Laboratory of Animal Genetics, Breeding and Reproduction, Ministry of Agriculture, China Agricultural University, Beijing, 100193 People’s Republic of China; Institute of Apicultural Research, Chinese Academy of Agricultural Sciences, Beijing, 100093 People’s Republic of China

**Keywords:** Expression, Implantation, Litter size, Pigs, SNPs

## Abstract

**Background:**

Embryonic mortality during the period of implantation strongly affects litter size in pigs. Progesterone receptor (*PGR*) paracrine signaling has been recognized to play a significant role in embryonic implantation. *IHH*, *NR2F2*, *BMP2*, *FKBP4* and *HAND2* were proved to involve in *PGR* paracrine signaling. The objective of this study was to evaluate the expression of *IHH*, *NR2F2*, *BMP2*, *FKBP4* and *HAND2* in endometrium of pregnant sows and to further investigate these genes’ effect on litter size in pigs. Real-time PCR, western blot and immunostaining were used to study target genes/proteins expression in endometrium in pigs. RFLP-PCR was used to detect single nucleotide polymorphisms (SNPs) of target genes.

**Results:**

The results showed that the mRNA and protein expression levels of *IHH*, *NR2F2* and *BMP2* were up-regulated during implantation period (*P* < 0.05 or *P* < 0.01). All target proteins were mainly observed in luminal epithelium and glandular epithelium. Interestingly, the staining of NR2F2 and HAND2 was also strong in stroma. SNPs detection revealed that there was a -204C > A mutation in promoter region of *NR2F2* gene. Three genotypes were found in Large White, Landrace and Duroc sows. A total of 1847 litter records from 625 sows genotyped at *NR2F2* gene were used to analyze the total number born (TNB) and number born alive (NBA). The study of the effect on litter size suggested that sows with genotype CC tend to have higher litter size.

**Conclusions:**

These results showed the expression patterns of genes/proteins involved in *PGR* paracrine signaling over implantation time. And the candidate gene for litter size was identified from genes involved in this signaling. This study could be a resource for further studies to identify the roles of these genes for embryonic implantation in pigs.

**Electronic supplementary material:**

The online version of this article (doi:10.1186/s40104-016-0090-z) contains supplementary material, which is available to authorized users.

## Background

Most reproductive traits are complex in terms of their genetic architecture [1]. Litter size is one of the most important economical traits in pig production. But as a quantitative trait, the heritability of litter size is low (0.1–0.15) [2]. Also litter size cannot be measured until the age of sexual maturity. However, these biological constraints can be potentially ameliorated by a better knowledge of the genetic regulation of litter size, which will lead to new tools to implement gene and/or marker assisted selection [3].

Implantation process is one of the important factors that affect litter size in pigs, owing to the high embryonic mortality during this stage. Due to the significant role that progesterone receptor (*PGR*) plays in pregnancy [4–7], paracrine signaling initiated by *PGR* within the uterine microenvironment during implantation period promotes implantation of conceptus and also promotes the development and maintenance of gestation [8, 9]. It has been proved that during early stage of pregnancy the function of *PGR* can be successfully transmitted through *HH–NR2F2* signaling axis. Indian hedgehog (*IHH*), which was identified as an acute *PGR* target gene [10], is a known member of the hedgehog (*HH*) signaling pathway. The HH signaling pathway has been demonstrated to be critical for embryonic development, which operates in an epithelial to mesenchymal manner within the uterus (reviewed in [11]). *NR2F2* (nuclear receptor subfamily 2, group F, member 2) has been identified to be a critical regulator in cell differentiation and tissue development as well as angiogenesis and metabolism (reviewed in [12]). *IHH* and *NR2F2* interaction works as *HH–NR2F2* axis, which plays a role in transducing an epithelial to stromal signal that initiates embryonic implantation and subsequently decidualization. *BMP2* (bone morphogenetic protein 2) and *FKBP4* (FK506 binding protein 4) worked as down-stream target genes of *HH-NR2F2* axis, which were necessary and sufficient for implantation and decidualization. *BMP2* acts via a paracrine mechanism to initiate decidualization after embryonic implantation, and also plays a fundamental role in preparing the epithelium for implantation through the regulation of Fkbps and Wnt ligands. *HAND2* is a basic helix-loop-helix (bHLH) transcription factor and a known downstream target of *PGR. HAND2* is a critical mediator between active paracrine signaling by *PGR* signaling and the inhibition of estrogen-induced proliferation within the epithelium, which is critical for embryonic implantation.

Therefore, *PGR* paracrine signaling is critical for embryonic implantation. Porcine embryos begin to attach to the uterus on pregnancy day 13 and 14, and implantation completes from pregnancy day 18 to day 24 [13]. In this research, we detected the expression level of the genes/proteins involved in *PGR* paracrine signaling, including *IHH*, *NR2F2*, *BMP2*, *FKBP4* and *HAND2*, in the endometrium on d 13, 18 and 24 of gestation in pigs. SNPs of these genes were detected and the association between the polymorphism and litter size in Large White, Landrace and Duroc pigs was analyzed. The results will provide information towards a better understanding of *PGR* paracrine signaling, which regulates implantation and subsequently affect litter size in pigs.

## Methods

### Animal materials

The Animal Care and Use Committee of China Agricultural University reviewed and approved the experimental protocol used in this study (Code: SYXK (Jing) 2009-0030). Multiparous Large White sows (5^th^ parity) were observed daily for standing heat in the presence of a boar. The sows of the pregnant groups (three groups, three sows each group) were inseminated twice, 12 h and 24 h after heat detection, respectively [14]. The sows of the non-pregnant group (three sows) were treated with inactivated sperm from the same boar [14]. Pregnant sows were slaughtered by electrocution on d 13, 18 and 24 after insemination. Samples of the endometrium attachment sites and inter-sites were taken. Samples were taken from three locations of each uterine horn: proximal (the end, close to the ovaries), medial, and distal (next to the corpus uteri) [14]. Non-pregnant sows were slaughtered on d 13 after insemination. Samples were taken from the comparable locations. Endometrial tissue sampling was carried out according to the procedure of Lord, with minor modifications [15]. The samples used for real time PCR and western-blot were collected immediately, snap frozen in liquid nitrogen and stored at −80 °C. The samples used for immunohistochemistry were collected and placed in a tube containing pre-cooling paraformaldehyde solution (4 %, pH = 7.4) and placed on a rocker overnight for fixation of the tissue. Once the period of fixation was finished, the tissue was rinsed in PBS, and then processed through a series of ethanol washes to displace the water. Then the tissue was infiltrated with and embedded in paraffin. Paraffin-embedded tissues were sliced at 5 μm thickness using a microtome (Leica2016, Germany).

Animals used to identify candidate genes for litter size were from Beijing Huadu Swine Breeding Company LTD. All sows were reared and feed in the same condition. Ear tissue samples of 625 Large White, Landrace and Duroc sows were collected in centrifuge tubes (1.5 mL) with 70 % ethanol and stored at 4 °C until DNA extraction. DNA was extracted by phenol and chloroforms (1:1) extraction. There are eight sire families in Large White, eight sire families in Landrace, and seven sire families in Duroc sows. 1847 litters’ records were used for statistical analysis. Litter size records such as total number born (TNB) and number born alive (NBA) were recorded by parity.

### RNA isolation and real time quantitative PCR

Trizol reagent (Invitrogen, Carlsbad, CA, USA) was used to extract total RNA, according to the manufacturer’s instructions. For each animal, total RNA consisted of a mix of an equal quantity of total RNA from three locations of each uterine horn: proximal (the end, close to the ovaries), medial, and distal (next to the corpus uteri).

For each sample, first strand cDNA was synthesized using 1 μg of total RNA. M-MLV FIRST STRAND KIT (Invitrogen, Shanghai, China) and oligo (dT)18 primer were used in a total of 20 μL reverse transcription reaction following the supplier’s instruction. Transcript specific primer pairs (see Additional file 1: Table S1) were designed with Oligo 6.0 software. Standard PCRs on cDNA were carried out to verify amplification sizes. Transcript quantification was performed using SYBR Green mix (Roche Diagnostics GmbH, Roche Applied Science, Mannheim, Germany) in a Roche LightCylcer 480 (Roche Diagnostics GmbH, Roche Applied Science, Mannheim, Germany). The RT-PCR reactions were prepared in a total volume of 20 μL containing 5 μL of cDNA (50 ng, 1:100 dilution), 10 μL of SYBR Green mix, 3 μL water which contained in the kit and 0.02 μmol/L of both forward and reverse gene specific primers. Glyceraldehyde-3-phosphate dehydrogenase (*GAPDH*) served as the internal reference gene. Cycling conditions were 95 °C for 10 min, followed by 45 cycles of 95 °C (10 s) and 60 °C (10 s) where the fluorescence was acquired. Finally, a dissociation curve to test PCR specificity was generated by one cycle at 95 °C (10s) followed by 60 °C (1 min) and ramp up to 95 °C with acquired fluorescence during the ramp to 0.2 °C/s. PCR efficiency of each gene was estimated by standard curve calculation using four points of cDNA serial dilutions. Ct values were transformed to quantities using the comparative Ct method, setting the relative quantities of non-pregnant group for each gene to 1 (*Qty* = 10-ΔCt/slope). Data normalization was carried out using *GAPDH* as the reference gene. Comparisons of genes expression levels were done using a *t*-test.

### Western-blot

Frozen sections of endometrial samples were prepared and western blotting was performed as previously described with minor modification [16]. Tissues protein was extracted (0.05 mol/L Tris–HCl, NaCl 8.76 mg/mL, 1 % TritonX-100 and 100 μg/mL PMSF) (Sunbio, China) by vortex meter (Kylinbell, China). Total protein concentrations were detected using the BCA Protein Assay Kit (Sunbio, China) according to the manufacturer’s recommendations.

Sample 80–120 μg was separated in a 10 % Tris–HCl polyacrylamide gel in electrophoresis system (Liuyi, China), and protein from the gel was transferred onto a single PVDF membrane (BioRad, USA). After rinsed in TBST for 5 min at room temperature (RT), the membrane was soaked in 5 % skim milk (in TBST) for 1 h. Next, the membrane was immerged into specific dilution (IHH, Santa Cruz Biotechnology, Inc., sc-13088, 1:100; NR2F2, Abcam (Hong Kong) Ltd., ab50487, 1:100; BMP2, Abcam (Hong Kong) Ltd., ab14933, 1:100; FKBP4, Abcam (Hong Kong) Ltd., ab97306, 1:150; HAND2, Biobyt, orb36304, 1:100;β-Actin 1:200) of the primary antibody at 4 °C overnight. After rinsed in TBST for 5 min three times at RT, the membrane was immerged into 1:1000 dilution of the secondary antibody (HRP) (Santa Cruz, USA) for 1 h, and then rinsed in TBST for 5 min three times at RT. Finally, the membrane was colored using the DAB kit (Invitrogen, USA) and exposed using Chemiluminescence Detection Kit for HRP (Sunbio, China). Scanned images were quantified using Image J analysis software.

### Immunohistochemistry

Sows endometrial slides were subjected to immunohistochemical analysis with immunostaining kit, Histostain-Plus Mouse Primary (Invitrogen, USA) according to the manufacturer’s recommendations. After being washed in PBS, the sections were incubated with 10 % horse serum (Invitrogen, USA) at RT for 30 min. The washed sections were then reacted with primary antibodies (rabbit polyclonal to IHH, Santa Cruz Biotechnology, Inc., sc-13088; rabbit polyclonal to NR2F2, Abcam (Hong Kong) Ltd., ab50487; rabbit polyclonal to BMP2, Abcam (Hong Kong) Ltd., ab14933; rabbit polyclonal to FKBP4, Abcam (Hong Kong) Ltd., ab97306; rabbit polyclonal to HAND2, Biobyt, orb36304; mouse monoclonal toβ-Actin, Santa Cruz Biotechnology, Inc., sc-81178) at 4 °C overnight. Followed by incubation with biotinylated second antibody (Invitrogen, USA) at 37 °C for 25 min, and after being washed in PBS for 15 min three times, the sections were incubated with streptavidin-peroxidase (HRP) (Invitrogen, USA) at 37 °C for 25 min. Finally, the slides were washed with PBS and stained with DAB kit (Invitrogen, USA). After being washed fully with water for 5 min, the slides were stained with hematoxylin and eosin, and then examined by microscope (BH2, Olympus). Instead of primary antibodies, PBS was used as a negative control. Endometrial tissues of non-pregnant sows were used as positive control [17]. ImagePro Plus software was used to measure the level of staining. The gray value of the portion of the picture without tissue was set as 0 to correct the background. Scoring of staining was carried out according to the procedure of Constantine A. Axiotis (1991), with minor modifications [18]. Expression of target protein was determined by assessing the staining intensity and the percentage of stained cells. The staining intensity was rated as follows: weak staining (score = 1), moderate staining (score = 2), strong staining (score = 3). The percentages of positive cells was calculated using ImagePro plus. This formula was used to calculated the final score: ∑(percentage of positive cells)* (score of positive staining). Average of five different areas per picture was recorded. According to the final score, the protein expressed as follows: <1.0, weak, 1.0–1.5, moderate; >1.5, strong.

### Detection of SNPs and litter size association analysis

DNA was extracted by phenol and chloroforms (1:1) standard techniques. 18 PCR primer pairs (see Additional file 2: Table S2) were designed to detect SNPs of target genes. PCR amplifications were carried out on an Eppendorf Mastercycler gradient 5331 PCR System (Eppendorf, Germany). The polymerase chain reaction amplification was performed using 50–100 ng of genomic DNA, 25 μL Taq PCR MasterMix (Taq DNA Polymerase: 0.05 units/μL; MgCl_2_: 4 mM/μL; dNTPs: 0.4 mM/μL), 10 pM of each primers in a 50 μL final volume. All reagents were collected from the National Laboratories for Agrobiotechnology, China Agricultural University. The following conditions of PCR amplification were used: a denaturation step at 95 °C for 4 min, 30 cycles at 95 °C for 30 s, 52 °C ~ 55 °C for 30 s, and 72 °C for 30 s ~ 1 min 30 s, a final extension step of 72 °C for 10 min. Amplified fragments were separated by 1.5 % agarose gel electrophoresis (AGE).

Using pooled DNA amplification and sequencing, several mutations were found. Mutation −204C > A in promoter region of *NR2F2* gene caused the deletion of transcription factor binding sites (TFBS) CREB (cAMP-response-element-binding protein).

*NR2F2* was selected to be the candidate gene for litter size based on its mRNA/protein expression level during embryonic implantation period and the mutation found in promoter region. PCR- Restriction fragment length polymorphism (PCR-RFLP) was used to detect different genotypes. *Hae*III (NEB R0108L, BioLabs Inc.) was used. The PCR products of three genotypes were random selected and sequenced to validate the results.

Alleles and genotypes frequencies of *NR2F2* were calculated from the 625 sows, respectively. GLM procedure of SAS 8.02 software was used to compute the least square means of TNB and NBA. According to the analysis, the effect of sire and dam on litter size was not significant, so the following linear model was used to analyze the genotype effect of *NR2F2*.

$$ \mathrm{Yijkl}=\mu +\mathrm{HYSi}+\mathrm{P}\mathrm{j}+\mathrm{G}\mathrm{k}+\mathrm{eijkl} $$

Where Y_ijkl_ is the traits of TNB and NBA, *μ* is the overall mean, HYS_i_ is the effect of herd-year-season (*i* = 1 to 52), P_j_ is the effect of parity (*j* = 1, 2, ≥3 and all parities), G_k_ is the effect of genotype (*k* = 1 to 3) and e_ijkl_ is the random residual. The data was analyzed separately for the first parity, the second parity, the third and following parities, and all parities. The additive effect and the dominant effect were calculated according to the methods of Rothschild et al. [19].

## Results

### mRNA expression in porcine endometrium

The effect of the day of pregnancy on mRNA expression of *IHH, NR2F2, BMP2, FKBP4 and HAND2* in sows’ endometrium during implantation period was shown in Table 1. In pregnant sows, the expression of *IHH* was significantly higher than that of non-pregnant sows on d 18 and d 24 of pregnancy (*P* < 0.05) (Table 1). The expression of *IHH* in attachment sites showed an uptrend. This was consistent with the expression of *NR2F2* which was significantly up-regulated during implantation time.

**Table 1:** The mRNA level of target genes in the endometrium during implantation (M ± S.D.)

Target	Non-pregnant	D 13 of pregnancy		D 18 of pregnancy		D 24 of pregnancy	
		Attachment sites	Inter-sites	Attachment sites	Inter-sites	At sites	Inter-sites
*PGR*	1.01 ± 0.25^Aa^	−2.33 ± 0.01	−1.31 ± 0.21	−2.60 ± 0.21	−2.12 ± 0.16	−10.34 ± 0.16^B^	−3.94 ± 0.06^b^
*IHH*	1.05 ± 0.33^a^	1.34 ± 0.34	2.67 ± 0.71^b^	2.12 ± 0.71^b^	3.92 ± 1.48^b^	3.48 ± 1.58^b^	2.60 ± 0.67^b^
*NR2F2*	1.02 ± 0.18^A^	3.80 ± 0.91^B^	4.77 ± 0.99 ^B^	4.42 ± 0.17 ^B^	5.32 ± 0.73 ^B^	6.18 ± 1.75 ^B^	2.18 ± 0.64
*BMP2*	1.02 ± 0.18^Aa^	2.41 ± 0.19 ^B^	3.61 ± 0.50 ^B^	4.54 ± 0.94 ^B^	4.57 ± 0.97 ^B^	3.61 ± 1.52 ^b^	3.90 ± 1.70 ^b^
*FKBP4*	1.02 ± 0.21 ^A^	1.79 ± 0.39	1.50 ± 0.52	0.63 ± 0.19	0.99 ± 0.15	0.35 ± 0.08 ^B^	0.76 ± 0.21
*HAND2*	1.07 ± 0.14 ^a^	1.83 ± 0.33	2.09 ± 0.50	2.93 ± 0.83 ^b^	2.98 ± 0.87 ^b^	1.08 ± 0.16	1.11 ± 0.16

a, b *P* < 0.05, A, B *P* < 0.01

The expression of *BMP2* was significantly up-regulated (*P* < 0.05 or *P* < 0.01) during implantation time (Table 1), which was consistent with *IHH* and *NR2F2*. For *FKBP4*, at attachment sites, the expression of *FKBP4* was significantly down-regulated on d 24 of pregnancy (*P* < 0.01) (Table 1). The expression of *HAND2* was the highest on d 18 of pregnancy (*P* < 0.05) (Table 1).

### Protein expression in porcine endometrium

**Fig. 1:**
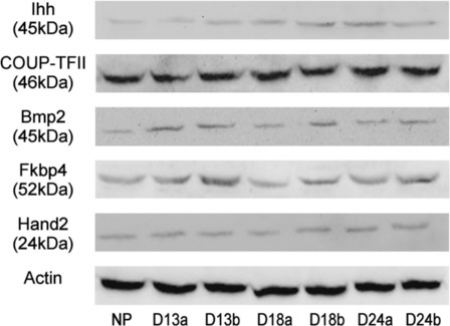
The protein relative abundance of target proteins in endometrium of sows. Note: NP, endometrium of non-pregnant sows; D13a, endometrial attachment sites on d 13 of gestation; D13b, the endometrial inter-sites on d 13 of gestation; D18a, endometrial attachment sites on d 18 of gestation; D13b, the endometrial inter-sites on d 18 of gestation; D24a, endometrial attachment sites on d 24 of gestation; D24b, the endometrial inter-sites on d 24 of gestation

The protein expressions of IHH, NR2F2, BMP2, FKBP4 and HAND2 in the porcine endometrium during the embryonic implantation period were shown in Fig. 1 and Table 2. The protein expression of IHH was significantly up-regulated on d 18 and d 24 of pregnancy (*P* < 0.05 or *P* < 0.01) (Fig. 1 and Table 2), which was similar to its mRNA expression. The protein expression of BMP2 was higher on d 13 of pregnancy (*P* < 0.05) (Fig. 1 and Table 2). For the protein expression of FKBP4, there was not significantly difference between pregnant groups and non-pregnant group (Fig. 1 and Table 2), which was not consistent with its mRNA expression pattern. The protein expression of HAND2 was higher in pregnant sows (*P* < 0.01) (Fig. 1 and Table 2), except at attachment sites on d 18 of pregnancy.

**Table 2:** The protein relative abundance of target proteins in endometrium of sows

Target	Non-pregnant	D 13 of pregnancy		D 18 of pregnancy		D 24 of pregnancy	
		Attachment sites	Inter-sites	Attachment sites	Inter-sites	Attachment sites	Inter-sites
IHH	0.28 ± 0.10^Aa^	0.33 ± 0.15	0.48 ± 0.11	0.48 ± 0.11^b^	1.00 ± 0.02^B^	1.03 ± 0.21^B^	0.72 ± 0.03^B^
NR2F2	0.89 ± 0.08	0.71 ± 0.05	0.99 ± 0.10	1.09 ± 0.02	1.15 ± 0.06	1.16 ± 0.07	1.11 ± 0.06
BMP2	0.37 ± 0.14^a^	0.77 ± 0.13^b^	0.58 ± 0.01^b^	0.38 ± 0.10	0.44 ± 0.11	0.33 ± 0.19	0.40 ± 0.21
FKBP4	0.57 ± 0.14	0.66 ± 0.16	1.00 ± 0.20	0.45 ± 0.19	0.66 ± 0.21	0.63 ± 0.18	0.84 ± 0.16
HAND2	0.61 ± 0.03^A^	0.73 ± 0.03^B^	0.71 ± 0.01^B^	0.57 ± 0.06	0.78 ± 0.01^B^	0.82 ± 0.03^B^	0.87 ± 0.05^B^

a, b *P* < 0.05, A, B *P* < 0.01

### Protein localization in porcine endometrium

**Fig. 2:**
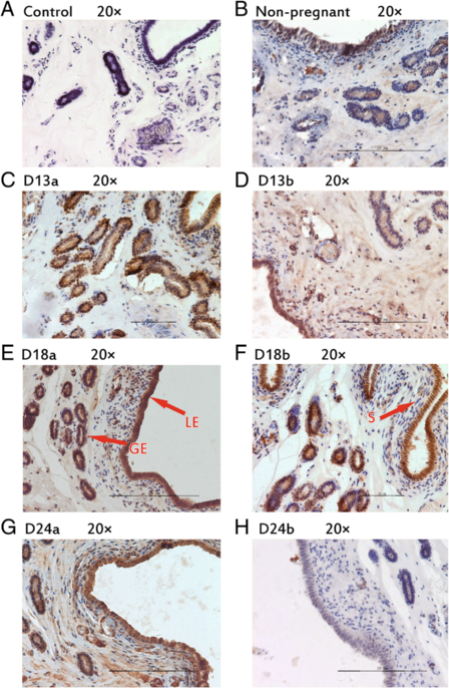
Immunhistochemical localization of IHH in pig uterus. GE = glandular epithelium; LE = luminal epithelium; S = stroma. **a** Negative control; **b** Immunohistochemical staining of non-pregnanct sows uterus with IHH antibody; **c** Immunohistochemical staining of porcine uterus attachment site with IHH antibody on d 13 of pregnancy; **d** Immunohistochemical staining of porcine uterus inter-site with IHH antibody on d 13 of pregnancy; **e** Immunohistochemical staining of porcine uterus attachment site with IHH antibody on d 18 of pregnancy; **f** Immunohistochemical staining of porcine uterus inter-site with IHH antibody on d 18 of pregnancy; **g** Immunohistochemical staining of porcine uterus attachment site with IHH antibody on d 24 of pregnancy; **h** Immunohistochemical staining of porcine uterus inter-site with IHH antibody on d 24 of pregnancy

**Fig. 3:**
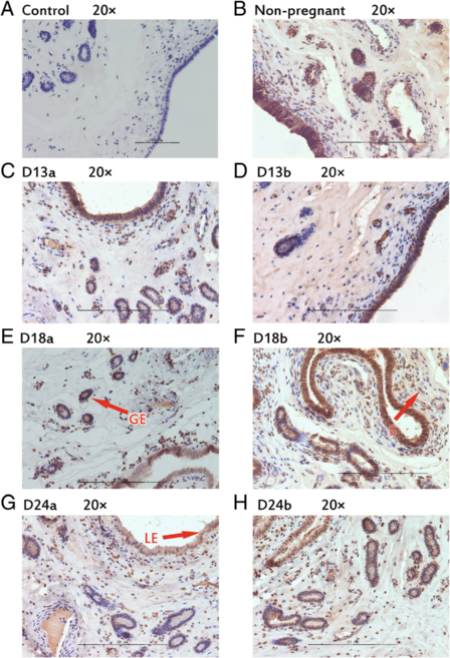
Immunhistochemical localization of NR2F2 in pig uterus. GE = glandular epithelium; LE = luminal epithelium; S = stroma. **a** Negative control; **b** Immunohistochemical staining of non-pregnanct sows uterus with NR2F2 antibody; **c** Immunohistochemical staining of porcine uterus attachment site with NR2F2 antibody on d 13 of pregnancy; **d** Immunohistochemical staining of porcine uterus inter-site with NR2F2 antibody on d 13 of pregnancy; **e** Immunohistochemical staining of porcine uterus attachment site with NR2F2 antibody on d 18 of pregnancy; **f** Immunohistochemical staining of porcine uterus inter-site with NR2F2 antibody on d 18 of pregnancy; **g** Immunohistochemical staining of porcine uterus attachment site with NR2F2 antibody on d 24 of pregnancy; **h** Immunohistochemical staining of porcine uterus inter-site with NR2F2 antibody on d 24 of pregnancy

**Fig. 4:**
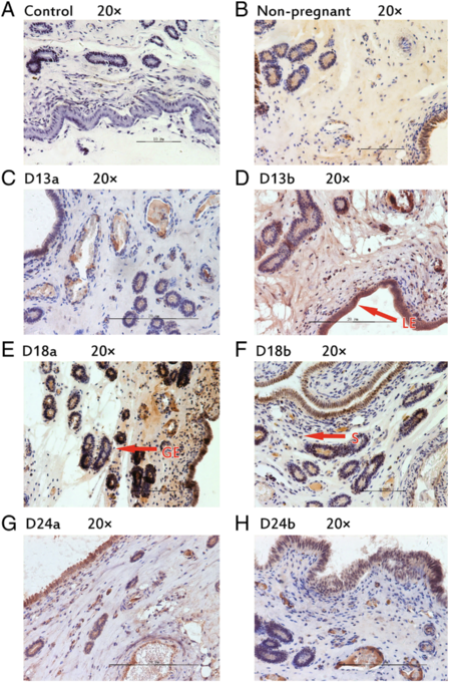
Immunhistochemical localization of BMP2 in pig uterus. GE = glandular epithelium; LE = luminal epithelium; S = stroma. **a** Negative control; **b** Immunohistochemical staining of non-pregnanct sows uterus with BMP2 antibody; **c** Immunohistochemical staining of porcine uterus attachment site with BMP2 antibody on d 13 of pregnancy; **d** Immunohistochemical staining of porcine uterus inter-site with BMP2 antibody on d 13 of pregnancy; **e** Immunohistochemical staining of porcine uterus attachment site with BMP2 antibody on d 18 of pregnancy; **f** Immunohistochemical staining of porcine uterus inter-site with BMP2 antibody on d 18 of pregnancy; **g** Immunohistochemical staining of porcine uterus attachment site with BMP2 antibody on d 24 of pregnancy; **h** Immunohistochemical staining of porcine uterus inter-site with BMP2 antibody on d 24 of pregnancy

**Fig. 5:**
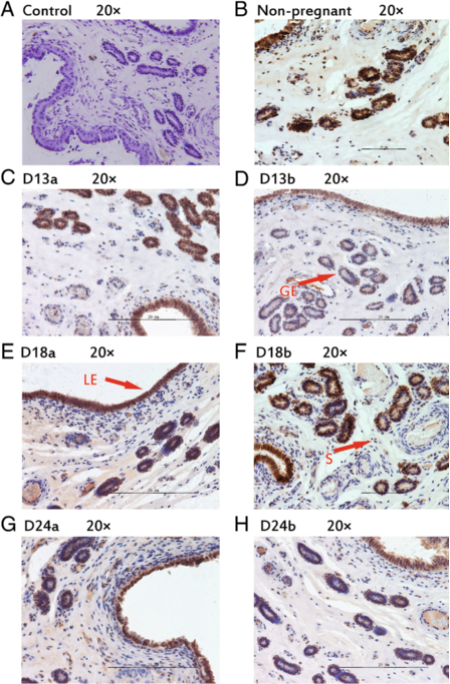
Immunhistochemical localization of FKBP4 in pig uterus. GE = glandular epithelium; LE = luminal epithelium; S = stroma. **a** Negative control; **b** Immunohistochemical staining of non-pregnanct sows uterus with FKBP4 antibody; **c** Immunohistochemical staining of porcine uterus attachment site with FKBP4 antibody on d 13 of pregnancy; **d** Immunohistochemical staining of porcine uterus inter-site with FKBP4 antibody on d 13 of pregnancy; **e** Immunohistochemical staining of porcine uterus attachment site with FKBP4 antibody on d 18 of pregnancy; **f** Immunohistochemical staining of porcine uterus inter-site with FKBP4 antibody on d 18 of pregnancy; **g** Immunohistochemical staining of porcine uterus attachment site with FKBP4 antibody on d 24 of pregnancy; **h** Immunohistochemical staining of porcine uterus inter-site with FKBP4 antibody on d 24 of pregnancy

**Fig. 6:**
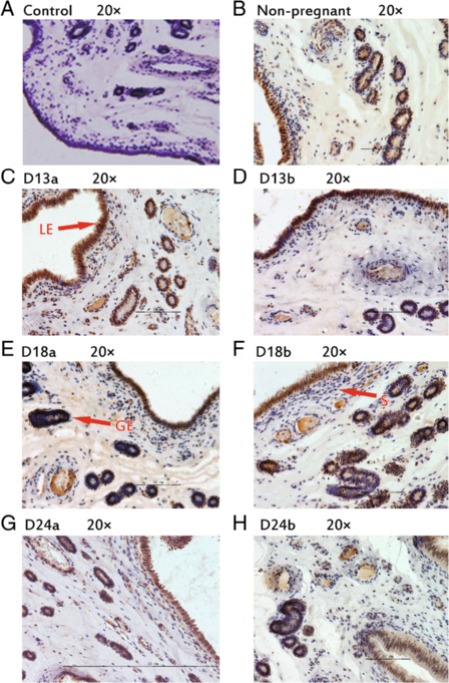
Immunhistochemical localization of HAND2 in pig uterus. GE = glandular epithelium; LE = luminal epithelium; S = stroma. **a** Negative control; **b** Immunohistochemical staining of non-pregnanct sows uterus with HAND2 antibody; **c** Immunohistochemical staining of porcine uterus attachment site with HAND2 antibody on d 13 of pregnancy; **d** Immunohistochemical staining of porcine uterus inter-site with HAND2 antibody on d 13 of pregnancy; **e** Immunohistochemical staining of porcine uterus attachment site with HAND2 antibody on d 18 of pregnancy; **f** Immunohistochemical staining of porcine uterus inter-site with HAND2 antibody on d 18 of pregnancy; **g** Immunohistochemical staining of porcine uterus attachment site with HAND2 antibody on d 24 of pregnancy; **h** Immunohistochemical staining of porcine uterus inter-site with HAND2 antibody on d 24 of pregnancy

During implantation period, IHH, NR2F2, BMP2, FKBP4 and HAND2 were observed in luminal epithelium and glandular epithelium (Figs. 2, 3, 4, 5, 6). In stroma, the staining of BMP2 and FKBP4 were weak, but the staining of NR2F2 and HAND2 was strong (Figs. 2, 3, 4, 5, 6). The result was summarized in Table 3.

**Table 3:** The expression of different position of target proteins in endometrium of sows

Target	Non-pregnant			D 13 of pregnancy						D 18 of pregnancy						D 24 of pregnancy					
				Attachment sites			Inter-sites			Attachment sites			Inter-sites			Attachment sites			Inter-sites		
	GE	LE	S	GE	LE	S	GE	LE	S	GE	LE	S	GE	LE	S	GE	LE	S	GE	LE	S
IHH	++	++	±	++	++	+	++	++	±	++	++	+	++	++	+	++	++	+	++	++	±
NR2F2	++	++	±	++	++	+	++	++	+	++	++	++	++	++	++	++	++	++	++	++	++
BMP2	++	++	±	++	++	±	++	++	±	++	++	+	++	++	±	++	++	+	++	++	±
FKBP4	++	++	±	++	++	±	++	++	±	++	++	±	++	++	±	++	++	±	++	++	±
HAND2	++	++	±	++	++	+	++	++	+	++	++	+	++	++	+	++	++	+	++	++	+

*GE* glandular epithelium; *LE* luminal epithelium; *S* stroma

± weak; +moderate; ++strong

### Detection of SNPs of target genes and association analysis

**Fig. 7:**
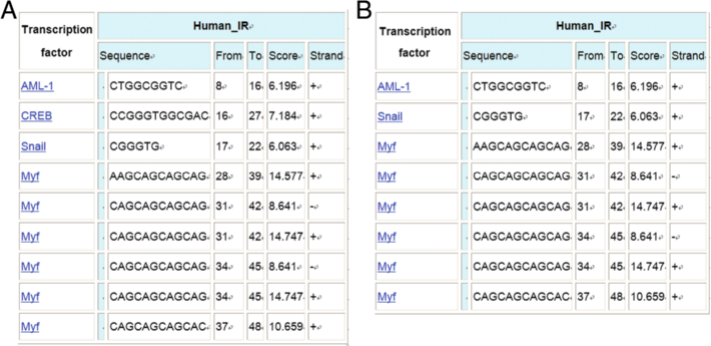
Change of transcription factor caused by mutation. **a** C at 204 bp; **b** A at 204 bp

After analysis samples of 625 sows, several mutations were found (Table 4). Mutation -204C > A in promoter region of *NR2F2* gene was found, and this mutation caused the deletion of TFBS CREB (Fig. 7). Synonymous mutation 9619G > A in exon 3 of *BMP2* gene was found (Table 4). Seven mutations in *FKBP4* gene were found, but no one is missense mutation (Table 4).

**Table 4:** Location and type of nucleotide mutation of target genes

Target	Location	Exon/Intron	Mutation	Type
*NR2F2*	−204 bp	5′-promoter	C > A	N
*BMP2*	9619 bp	Exon 3	G > A	Synonymous mutation
*FKBP4*	2198 bp	Intron 1	C > T	N
	2203 bp	Intron 1	G > A	N
	2375 bp	Intron 2	A > G	N
	2949 bp	Exon 3	A > T	Synonymous mutation
	6086 bp	Intron 8	C > T	N
	6163 bp	Exon 9	C > T	Synonymous mutation
	6233 bp	Exon 9	T > C	Synonymous mutation

**Fig. 8:**
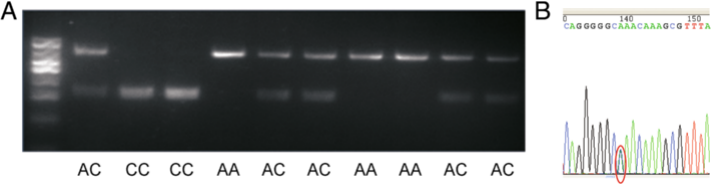
PCR-RFLP results of swine *NR2F2* gene and sequence image of the different genotypes. **a** Genotypes of the RFLP marker of PCR products; **b** Sequence image of mutation -204C > A

*NR2F2* was selected to be the candidate gene for litter size based on its mRNA/protein expression level during embryonic implantation period and the mutation found in promoter region. PCR-RFLP was used to detect different genotypes. The representative SNPs sequencing output for genotypes were shown in Fig. 8. The genotype frequencies and allele frequencies at each polymorphic locus in Large White, Landrace and Duroc sows were shown in Table 5. The genotype frequencies of AA, AC and CC in large white were 0.388, 0.414, and 0.198. In Landrace, the genotype frequencies were 0.088, 0.366, and 0.546. In Duroc, the genotype frequencies were 0.358, 0.433, and 0.208. None of the three breeds was found to be in Hardy-Weinberg equilibrium (HWE).

**Table 5:** Number of alleles (n), allele and genotype frequencies of *NR2F2*, observed heterozygosity (h)

Breed	Sows	Genotype distribution			Genotype frequencies			Allele frequencies		h
		AA	AC	CC	AA	AC	CC	A	C	
Large White	232	90	96	46	0.388	0.414	0.198	0.595	0.405	4.648
Landrace	273	24	100	149	0.088	0.366	0.546	0.271	0.729	1.458
Duroc	120	43	52	25	0.358	0.433	0.208	0.575	0.425	1.542

The data for TNB and NBA were observed for the first parity, the second parity, the third and the following parities and all parities. The least square means in Large White, Landrace and Duroc were shown in Tables 6, 7 and 8. In Large White, in the first parity, the sows with AA genotype had an advantage of 0.81 (*P* < 0.05) NBA per litter over the sows with CC genotype. In the second parity, the sows with CC genotype had an advantage of 1.76 (*P* < 0.01) and 1.56 (*P* < 0.01) TNB per litter over the sows with AA and AC, respectively. NBA of CC genotype were of 0.99 (*P* < 0.05) more piglets per litter than that of the AA genotype. In the third and following parities, NBA significantly increased for the CC genotype with 0.60 (*P* < 0.05) and 0.85 (*P* < 0.01) more piglets in comparison with the AA and AC genotype, respectively. In all parities, the sows with CC genotype had an advantage (*P* < 0.05) of 0.89 and 0.64 for TNB per litter over the AA and AC genotype sows, respectively. And NBA of CC genotype were of 0.97 (*P* < 0.01) and 0.88 (*P* < 0.01) more piglets per litter than that of the AA and AC genotype, respectively.

**Table 6:** Effects of the *NR2F2* polymorphism on total number born (TNB) and number born alive (NBA) in Large White (LS means ± S.E.)

Breed	Genotype	First parity			Second parity			Third to ninth parity			All parities		
		Litters	TNB	NBA	Litters	TNB	NBA	Litters	TNB	NBA	Litters	TNB	NBA
Large White	AA	90	11.12 ± 0.25	10.33 ± 0.23^a^	60	11.10 ± 0.40^A^	10.21 ± 0.35^a^	144	11.078 ± 0.40	10.09 ± 0.35^a^	294	10.99 ± 0.32^A^	10.23 ± 0.28^A^
	AC	96	11.43 ± 0.25	10.59 ± 0.24	66	11.30 ± 0.41^A^	10.01 ± 0.37	190	10.96 ± 0.40	9.84 ± 0.36^A^	352	11.24 ± 0.32^A^	10.32 ± 0.29^A^
	CC	46	11.72 ± 0.32	11.14 ± 0.31^b^	26	12.86 ± 0.55^B^	11.20 ± 0.49^b^	93	10.69 ± 0.46	10.69 ± 0.41^Bb^	165	11.88 ± 0.35^B^	11.20 ± 0.32^B^

Values with different superscripts show significant levels within columns: a, b *P* < 0.05, A, B *P* < 0.01

**Table 7:** Effects of the *NR2F2* polymorphism on total number born (TNB) and number born alive (NBA) in Landrace (LS means ± S.E.)

Breed	Genotype	First parity			Second parity			Third to ninth parity			All parities		
		Litters	TNB	NBA	Litters	TNB	NBA	Litters	TNB	NBA	Litters	TNB	NBA
Landrace	AA	24	11.02 ± 0.32	10.44 ± 0.31	11	10.33 ± 0.53	10.06 ± 0.47	15	10.68 ± 0.30	10.24 ± 0.33	50	10.62 ± 0.19^a^	10.13 ± 0.23
	AC	100	11.15 ± 0.25	10.54 ± 0.25	52	10.85 ± 0.39	10.25 ± 0.34	78	11.19 ± 0.23	10.79 ± 0.30	230	10.98 ± 0.15	10.44 ± 0.20
	CC	149	11.36 ± 0.27	10.75 ± 0.27	97	11.26 ± 0.45	10.76 ± 0.40	151	11.21 ± 0.26	10.85 ± 0.30	397	11.67 ± 0.16^b^	10.66 ± 0.21

Values with different superscripts show significant levels within columns: a, b *P* < 0.05, A, B *P* < 0.01

**Table 8:** Effects of the *NR2F2* polymorphism on total number born (TNB) and number born alive (NBA) in Duroc (LS means ± S.E.)

Breed	Genotype	First parity			Second parity			Third to ninth parity			All parities		
		Litters	TNB	NBA	Litters	TNB	NBA	Litters	TNB	NBA	Litters	TNB	NBA
Duroc	AA	43	10.43 ± 0.35	9.53 ± 0.37	21	10.88 ± 0.39^a^	9.87 ± 0.35^A^	58	9.68 ± 0.34^A^	9.26 ± 0.39^a^	122	10.02 ± 0.20	9.30 ± 0.24
	AC	52	10.54 ± 0.30	10.07 ± 0.32	36	11.12 ± 0.39	9.98 ± 0.35^A^	103	10.48 ± 0.25	9.97 ± 0.31	161	10.21 ± 0.16	9.63 ± 0.21
	CC	25	10.60 ± 0.43	10.10 ± 0.46	11	11.54 ± 0.45^b^	10.81 ± 0.40^B^	40	11.03 ± 0.46^B^	10.60 ± 0.4^b^	76	10.17 ± 0.26	9.47 ± 0.30

Values with different superscripts show significant levels within columns: a, b *P* < 0.05, A, B *P* < 0.01

In Landrace, in the third and following parities, the sows with CC genotype had an advantage of 0.53 for TNB and 0.61 for NBA per litter over the sows with AA genotype, and 0.53 for TNB over the sows with AC genotype, but not significantly. In all parities, TNB of genotype CC was 1.05 (*P* < 0.05) piglets higher than that of the AA genotype. And the sows with the CC genotype had an advantage of 0.53 and 0.22 for NBA per litter over the sows with AA and AC, but not significantly.

In Duroc, in the second parity, the sows with the CC genotype had an advantage of 0.66 piglets (*P* < 0.05) for TNB and 1.34 (*P* < 0.05) piglets for NBA per litter over the sows with AA genotype. In the third and following parities, the sows with the CC genotype had an advantage of 1.35 piglets (*P* < 0.01) for TNB and 1.34 (*P* < 0.05) for NBA per litter over the sows with AA genotype.

## Discussion

### Expression of genes participated in paracrine signaling in sows endometrium

The embryonic peri-implantation time of pigs is especially longer. During the peri-implantation period of pregnancy, uterine LE and conceptus trophectoderm develop adhesion competency in synchrony to initiate the adhesion cascade within a restricted period of the uterine cycle termed the “window of receptivity” [20–22]. In pigs, this window is orchestrated through the actions of progesterone and estrogen to regulate locally produced cytokines, growth factors, cell surface glycoproteins, cell surface adhesion molecules, and extracellular matrix (ECM) proteins [23]. A fundamental paradox of early pregnancy is that cessation of expression of *PGR* and *ESR1* by uterine epithelia is a prerequisite for uterine receptivity to implantation, expression of genes by uterine epithelia and selective transport of molecules into the uterine lumen that support conceptus development. Thus, effects of *P4* are mediated via *PGR* expressed in uterine stromal and myometrial cells by stromal cell derived growth factors known as “progestamedins” [24, 25]. As previous indicated, progesterone down regulated the expression of *PGR* in the uterine epithelia of pigs after d 10 of pregnancy, immediately prior to the time when the endometrium becomes receptive to implantation [26–28]. In pigs, down-regulation of *PGR* in uterine epithelia is a prerequisite for the expression of genes for uterine secretions and transport of molecules into the uterine lumen that support conceptus development. Down-regulation of *PGR* is associated with down-regulation of mucin1 (*MUC1*), as well as up-regulation of the expression of secreted phosphoprotein 1 (*SPP1*) and insulin-like growth factor binding protein 1 (*IGFBP1*). During conceptus elongation and the early peri-implantation period, the endometrium increases the release of a number of growth factors and cytokines such as epidermal growth factor (*EGF*), insulin-like growth factor-1 (*IGF-1*), fibroblast growth factor 7 (*FGF7*), vascular endothelial growth factor (*VEGF*), interleukin 6 (*IL-6*), transforming growth factor beta (*TGFβ*), and leukemia inhibitory factor (*LIF*) [29, 30]. Some of these genes had been reported to have significant effect on litter size in pigs, such as *SPP1, VEGF, MUC1, LIF* et al [1, 31–33].

*PGR* paracrine signaling has been recognized to play a significant role in pregnancy in human and mouse, which have not been studied in pigs [5]. *IHH* is a progesterone receptor target activated within the epithelium which signals downstream to *NR2F2* in the stroma establishing the *HH–NR2F2* axis within the dual uterine compartments. Strong evidence exists to propose a role of a *HH–NR2F2* axis in the regulation of reproduction in human and mice [12, 34]. Identification of the signaling pathway from stroma to epithelium would aid in the understanding of how the stroma contributes to embryo implantation. Changes in endometrial transcriptome during early stages of conceptus attachment to uterine LE in previous study showed that *IHH* regulated significantly during pregnancy period in the pigs. In the present study, compared with non-pregnant sows, the mRNA and protein expression of *IHH* were up-regulated during implantation. The expression of *IHH* in bovine uterus had been studied. The result showed *IHH* is modulated by progesterone in bovine uterus, and may be required to be down-regulated to allow expression of genes that drive conceptus elongation in cattle [35]. In pigs, the conceptus elongated rapidly before d 13 of gestation, and the filamentous conceptus continue to elongate but slowly after d 13 of gestation. The expression of *IHH* did not show significantly changed at d 13 of pregnancy in our result. It may be because the conceptus elongate slowly after d 13 of pregnancy in pigs [36]. The expression of *NR2F2* was significantly up-regulated during implantation time and the expression in attachment sites showed an upward trend. This was consistent with previous study, which found *NR2F2* up-regulated in d 12 of gestation in Yorkshire pigs [37]. *NR2F2* was shown to activate hypoxia-inducible factor 1 alpha (*HIF-1α*) and *HIF-1* is an important mediator of estrogen-induced *VEGF* expression in the uterus [38, 39]. They thought that the expression of *NR2F2* is associated with greater activation of angiogenesis at the stage of implantation in the Yorkshire breed [37]. The expression of *IHH* and *NR2F2* were consistent with their functional role in embryonic implantation and also consistent with previous studies [40–44]. It was reported that *HH-NR2F2* axis can transmit the paracrine signaling by *PGR* from epithelium to stroma [42]. The protein localization of IHH in porcine endometrium showed that IHH mainly observed strongly in luminal epithelium and glandular epithelium. NR2F2 was especially observed strongly in stroma. This confirmed that *HH–NR2F2* axis was important in mediating the signal from epithelial to other effect or genes in the stroma.

*BMP2*, as a downstream gene of HH-NR2F2 axis, has demonstrated to be a critical effector for decidualization and the maintenance of pregnancy during post-implantation. *BMP2* likely acts as a paracrine signaling factor for the initiation of the proliferative response after embryonic implantation within the uterine stroma. In the present study, the mRNA expression of *BMP2* was significantly up-regulated during implantation time, which was consistent with the expression of *IHH* and *NR2F2*. In previous study, researchers found that *BMP2* and *BMP6* can significantly suppress progesterone production in pigs in vitro [45]. So this was consistent with our result, which showed *BMP2* up-regulated along with *PGR* down-regulated during implantation period. The protein expression of BMP2 was significantly up-regulated on d 13 of pregnancy, which demonstrated that BMP2 promotes implantation cooperated with IHH and NR2F2. But on d 18 and 24, the expression did not regulate significantly. It may be because decidualization did not happen in pigs.

*HAND2* was another downstream target of *PGR* [8]. In the stroma, *HAND2* plays an important role in the inhibition of the FGF pathway, a pathway known to be involved in the promotion of epithelial proliferation by estrogen signaling [8]. Therefore, *HAND2* is important to inhibit the estrogen-induced epithelial proliferation in the uterus [8]. The inhibition of epithelial proliferation by *PGR* signaling was possibly via *HH–NR2F2* axis. *HH–NR2F2* axis then activated *HAND2*, which caused the inhibition of estrogen signaling and subsequent allowance for proper embryonic implantation. In the present study, the expression of mRNA and protein of HAND2 were both up-regulated on d 13 of pregnancy. This may related with its inhibition of estrogen signaling, and further more promoted the positive role of *PGR* in implantation. In previous studies, *HAND2* had been detected up-regulated at implantation period and late gestation period in pigs [31, 46]. The researchers find *HAND2* related with receptivity of uterus and vascular development of placenta [31, 46]. The mRNA of *HAND2* was up-regulated on d 18 of pregnancy, but the protein expression was not. Maybe there is regulation mechanism at translation level, which needs further research. The protein localization in porcine endometrium showed that HAND2 observed strongly in luminal epithelium, glandular epithelium, and stroma. This indicated that *HAND2* played an important role in transmit the *PGR* signaling from epithelium to stroma.

### The variations of *NR2F2* and its association with litter size

Marker-assisted selection (MAS) in conjunction with traditional selection methods is most effective for the traits such as litter size, which are either expressed later in life, are sex-dependent, or are of low heritability [47]. The candidate gene approach has led to notable success in demonstrating reproduction-related genetic markers or major genes, such as *ESR*, *PRLR*, the erythropoietin receptor (*EPOR*) and so on [19, 48–50].

In the present study, we selected *NR2F2* as the candidate gene for litter size in pigs, due to its biological function and the interesting mutation. Three genotypes were found: AA, AC and CC. The association with litter size revealed that CC genotype is the favorable genotype. Through analysis using Consite database (http://consite.genereg.net/cgi-bin/consite?rm=t_input_single), the C → A mutation caused deletion of TFBS CREB (Fig. 7). CREB has been proved played an important role in activation of transcription and regulation of gene transcription [51, 52]. The deletion of CREB may affect the expression of *NR2F2* in porcine endometrium and stroma. The effect of *NR2F2* on litter size possibly associated with its expression in endometrium during embryonic implantation. This certainly will affect the signal of *PGR* from endometrium to stroma, in consideration of the PGR-IHH-NR2F2 axis. Subsequently, the embryonic implantation process and litter size was affected.

## Conclusions

In current research, the expression patterns of genes/proteins involved in *PGR* paracrine signaling over implantation time were studied. And candidate gene for litter size was identified from genes involved in this signaling. The present study could be a resource for further studies to identify the roles of these genes for embryonic implantation in pigs.

## Additional files

Additional file 1: Table S1. Primers used for Real-time PCR (RT-PCR). (DOCX 19 kb) Additional file 2: Table S2. Primer pairs and PCR conditions used for SNPs detection. (DOCX 20 kb)

## Abbreviations

AGE: agarose gel electrophoresis
CREB: cAMP-response-element-binding protein
D13a: endometrial attachment sites on day 13 of gestation
D13b: the endometrial inter-sites on day 13 of gestation
D13b: the endometrial inter-sites on day 18 of gestation
D18a: endometrial attachment sites on day 18 of gestation
D24a: endometrial attachment sites on day 24 of gestation
D24b: the endometrial inter-sites on day 24 of gestation
HWE: Hardy-Weinberg equilibrium
MAS: Marker-assisted selection
NBA: number born alive
NP: endometrium of non-pregnant sows
PCR-RFLP: PCR-Restriction fragment length polymorphism
PGR: progesterone receptor
TFBS: transcription factor binding sites
TNB: total number born
