# Supplementary material for: Expression of genes involved in progesterone receptor paracrine signaling and their effect on litter size in pigs
Source: J Anim Sci Biotechnol. 2016 May 25;7:31. doi: 10.1186/s40104-016-0090-z (PMC4881214; doi:10.1186/s40104-016-0090-z)
Supplement: Additional file 1: Table S1. — Primers used for Real-time PCR (RT-PCR). (DOCX 19 kb) [file 40104_2016_90_MOESM1_ESM.docx]

Table S1 Primers used for Real-time PCR (RT-PCR)

| Target | Forward/reverse primers | Annealing temperature | Length of amplification (bp) |
| --- | --- | --- | --- |
| *GAPDH* | F:GTCCACTGGTGTCTTCACGA | 60℃ | 154 bp |
|  | R:GCTGACGATCTTGAGGGAGT |  |  |
| *COUP-TFⅡ* | F: TGCCTGTGGTCTCTCTGATG | 60℃ | 117 bp |
|  | R: GGAAGGGAGGCGAAGCAAAA |  |  |
| *PGR* | F：CGGATTCAGAAGCCAGCCAGAG | 60℃ | 164 bp |
|  | R：TCCTCGTCCCAGCCCTCGGTCA |  |  |
| *Ihh* | F：GCTCACCCCTAACTACAATCCC | 60℃ | 168 bp |
|  | R：TCCTCGTCCCAGCCCTCGGTCA |  |  |
| *Bmp2* | F：TGGAGGCTCTTTCAATGGAC | 60℃ | 204 bp |
|  | R：CGAGGGCTGGGAAGAGGAGA |  |  |
| *Fkbp4* | F：TGGAGCAGAGCAGCATTGTGAAG | 60℃ | 145bp |
|  | R：TGTGCCTTCTGTGCGTCCTCGTT |  |  |
| *Hand2* | F: GGCGGAAATCAAGAAGACAG | 60℃ | 185bp |
|  | R: CTCTCCTCCTCCTCTTCCACCACCTCC |  |  |
