# Supplementary material for: Expression of genes involved in progesterone receptor paracrine signaling and their effect on litter size in pigs
Source: J Anim Sci Biotechnol. 2016 May 25;7:31. doi: 10.1186/s40104-016-0090-z (PMC4881214; doi:10.1186/s40104-016-0090-z)
Supplement: Additional file 2: Table S2. — Primer pairs and PCR conditions used for SNPs detection. (DOCX 20 kb) [file 40104_2016_90_MOESM2_ESM.docx]

Table S2 Primer pairs and PCR conditions used for SNPs detection

| Target | Forward/reverse primers | Annealing temperature | Length of amplification (bp) |
| --- | --- | --- | --- |
| Ihh-F1 | CCCGCCCCGCACCTGAGTCC | 63℃ | 585 bp |
| Ihh-R1 | CCAGCCAGTCAAGGAAAGGT |  |  |
| Ihh-F2 | CCTCAGGGACCCACACAGAC | 59℃ | 1418 bp |
| Ihh-R2 | CGGCGGGCCTCTTCACCTTCTT |  |  |
| Ihh-F3 | AACGCCTTCATGTCCCTTCTTC | 62℃ | 1301 bp |
| Ihh-R3 | CAAAAGGAAGAAAAGACCAAAA |  |  |
| COUP -F1 | GAGAGCGCCTAATACATGGGGA | 55℃ | 332 bp |
| COUP -R1 | GAGAAAAGTGAGAAAGGAGAGA |  |  |
| COUP-F21 | TGCCTGTGGTCTCTCTGATG | 55℃ | 962 bp |
| COUP-R21 | AGTGTTGTATGTGGTTCCTC |  |  |
| COUP-F22 | GATGATGGAAACGCACTTAC | 52℃ | 550 bp |
| COUP-R22 | ACACACAAAGACTCGACCAA |  |  |
| COUP -F3 | CCCAAGTTTTCAGTACAGAG | 55℃ | 1529 bp |
| COUP -R3 | ACACACAAAGACTCGACCAA |  |  |
| Bmp2-F1 | CGCGGCGGAGCTAGCACTGA | 60℃ | 610 bp |
| Bmp2-R1 | GTTCTGTTTACGGCTGGCGG |  |  |
| Bmp2-F2 | CTGGAGGAGGAGGCAAGTGG | 60℃ | 597 bp |
| Bmp2-R2 | ACGAGGCTTAGATAGGGATT |  |  |
| Bmp2-F3 | CAGATTGTCACCACTTGTCC | 60℃ | 899 bp |
| Bmp2-R3 | CCCACAACCCTCCACAACCA |  |  |
| Fkbp4-F1 | CGAGAAACGGCGCGATGAC | 64℃ | 268 bp |
| Fkbp4-R1 | GGGCCGTGGCTGCATGACCT |  |  |
| Fkbp4-F23 | GGTGTTTTGTCTCCAGTTTT | 60℃ | 1129 bp |
| Fkbp4-R23 | ACCTCCATCGCTGACCACTT |  |  |
| Fkbp4-F45 | TAATGGTCTCCTGAGGGTAA | 51℃ | 987 bp |
| Fkbp4-R45 | TGTAGCAGCCAAGAGTGAAA |  |  |
| Fkbp4-F678 | GAAGAGGTCAGGGAAGGAAG | 60℃ | 1025 bp |
| Fkbp4-R678 | AGGAGAGAACAATGAGGTGG |  |  |
| Fkbp4-F9 | TCTTCCCTCTCTGCTTCTTG | 52℃ | 626 bp |
| Fkbp4-R9 | GGAGAATGTAAGTTGGGAAG |  |  |
| Fkbp4-F10 | TGGGTGTGAGCAGGCAAGAA | 50℃ | 270 bp |
| Fkbp4-R10 | TACAAAACTAACAGGGTGGA |  |  |
| Hand2-F1 | GGCTGGTTGGGGGAAGAGA | 61℃ | 598 bp |
| Hand2-R1 | CAGGCCAAAGGGAAAGAGAC |  |  |
| Hand2-F2 | AAACGACTTGAAAACGGGTG | 60℃ | 454 bp |
